# DUSP1 protects against ischemic acute kidney injury through stabilizing mtDNA via interaction with JNK

**DOI:** 10.1038/s41419-023-06247-4

**Published:** 2023-11-07

**Authors:** Lang Shi, Hongchu Zha, Zhou Pan, Jiayi Wang, Yao Xia, Huimin Li, Hua Huang, Ruchi Yue, Zhixia Song, Jiefu Zhu

**Affiliations:** 1 Department of Nephrology, Renmin Hospital of Wuhan University, Wuhan, 430060 China; 2 Department of Nephrology, The First Clinical Medical College of Three Gorges University, Center People’s Hospital of Yichang, Yichang, Hubei 443000 China; 3 Department of Anesthesiology, the Xiangya Second Hospital, Central South University, Changsha, Hunan 410000 China; 4 Department of Urology, Renmin Hospital of Wuhan University, Wuhan, 430060 China; 5 Department of Organ Transplantation, Renmin Hospital of Wuhan University, Wuhan, 430060 China

**Keywords:** Acute kidney injury, Kidney

## Abstract

The mechanism underlying acute kidney injury (AKI) and AKI-to-Chronic kidney disease (CKD) transition remains unclear, but mitochondrial dysfunction may be a key driving factor. Literature reports suggest that dual-specificity phosphatase 1 (DUSP1) plays a critical role in maintaining mitochondrial function and structural integrity. In this study, ischemic Acute Kidney Injury (AKI) and post-ischemic fibrosis models were established by clamping the renal pedicle with different reperfusion times. To investigate the role of DUSP1, constitutional Dusp1 knockout mice and tubular-specific Sting knockout mice were used. Mitochondrial damage was assessed through electron microscopy observation, measurements of mitochondrial membrane potential, mtDNA release, and BAX translocation. We found that Dusp1 expression was significantly upregulated in human transplant kidney tissue and mouse AKI tissue. Dusp1 gene deletion exacerbated acute ischemic injury, post-ischemic renal fibrosis, and tubular mitochondrial dysfunction in mice. Mechanistically, DUSP1 could directly bind to JNK, and DUSP1 deficiency could lead to aberrant phosphorylation of JNK and BAX mitochondria translocation. BAX translocation promoted mitochondrial DNA (mtDNA) leakage and activated the cGAS-STING pathway. Inhibition of JNK or BAX could inhibit mtDNA leakage. Furthermore, STING knockout or JNK inhibition could significantly mitigate the adverse effects of DUSP1 deficiency in ischemic AKI model. Collectively, our findings suggest that DUSP1 is a regulator for the protective response during AKI. DUSP1 protects against AKI by preventing BAX-induced mtDNA leakage and blocking excessive activation of the cGAS-STING signaling axis through JNK dephosphorylation.

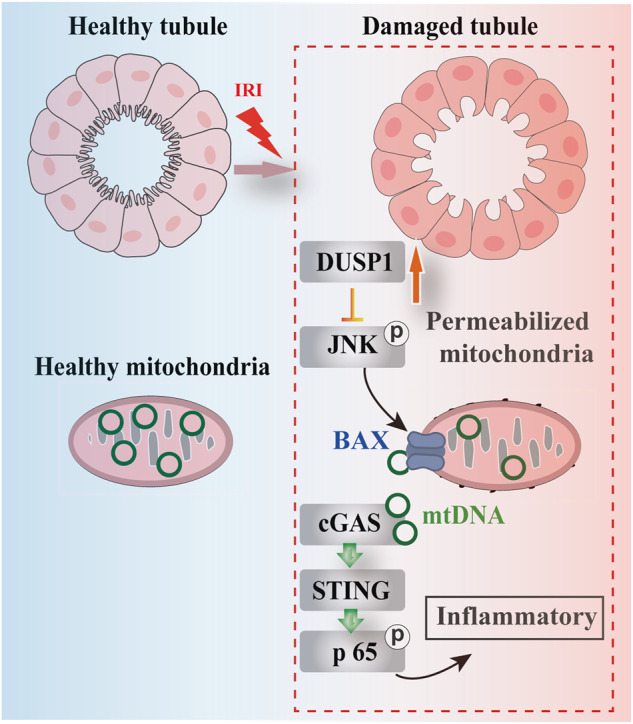

## Introduction

AKI is a clinical syndrome characterized by a sudden and sharp decline in renal function due to multiple factors, which has become a global clinical problem with high incidence and mortality [1, 2]. Although progress has been made in prevention and treatment, a significant proportion of AKI survivors cannot fully recover their kidney function and progress to CKD, even end-stage renal disease. Follow-up studies of hospitalized AKI patients have shown that 15–30% of them progress to CKD [3, 4]. Kidney fibrosis is one of the main pathological changes in CKD and a major mechanism leading to its progression and eventual renal failure [5, 6]. Due to the complex mechanisms underlying the transition of AKI to CKD, there is currently scarce specific treatment, but extensive clinical observations and animal studies have shown that mitochondrial dysfunction is involved in the pathogenesis of this process [7, 8].

The possible mechanisms of mitochondrial dysfunction mainly include mtDNA mutations, decreased mitochondrial biogenesis, imbalanced mitochondrial dynamics, and abnormally elevated oxidative stress [9–14]. The latest research has found that dysfunctional mitochondria can trigger inflammatory reactions by releasing mitochondrial DNA (mtDNA) [15–17]. Mitochondrial dysfunction and subsequent mtDNA-cGAS-STING pathway activation are key regulatory factors in kidney injury [18]. The cytoplasmic translocation of mtDNA and cGAS-STING pathway activation are also key factors leading to renal fibrosis in CKD mice [19, 20]. Although the pathological changes of mitochondria in AKI-CKD have been well studied, the pathways that induce mitochondrial dysfunction and lead to injury in renal tubular epithelial cells have not been fully determined.

DUSP1 encoded by the Dusp1 gene, is also known as mitogen-activated protein kinase phosphatase-1 (MKP1). DUSP1 can dephosphorylate multiple molecules involved in signal transduction pathways, such as p38 mitogen-activated protein kinase, JNK (c-Jun N-terminal kinase), and ERK (Extracellular signal-regulated kinases) [21–24]. Recent studies have shown that DUSP1 plays a critical role in maintaining mitochondrial function and structural integrity. DUSP1 could also improve the metabolism of mitochondria in inflammatory-induced cardiomyocytes by regulating mitosis and apoptosis [25]. Additionally, decreased expression of DUSP1 may lead to overactivation of MAPK and JNK signaling pathways, which can stimulate fibroblast proliferation and collagen synthesis, thereby promoting the progression of diabetic kidney disease and renal fibrosis [26]. Therefore, DUSP1 may play a regulatory role in mitochondrial dysfunction and kidney fibrosis. However, the role of DUSP1 in AKI and post-AKI fibrosis remains unknown.

RNA screening data showed that Dusp1 expression was upregulated in kidney tissues both from kidney transplant patients and mice with ischemic stimulation. This study aimed to investigate the role and mechanism of DUSP1 in ischemic AKI and post-AKI fibrosis, with a focus on mitochondrial dysfunction and mtDNA release in proximal tubular cells. Dusp1 deficiency exacerbated ischemia-induced renal dysfunction and mitochondrial dysfunction in renal cells. Mechanistically, DUSP1 could prevent BAX translocation to mitochondria via JNK and mtDNA release followed by BAX mitochondria accumulation. These findings support the protective role of DUSP1 in ischemia-driven renal function and mitochondrial dysfunction, providing new insights and approaches for the prevention and treatment of AKI.

## Materials and methods

### Animals

Dusp1 knockout (Dusp1-KO) mice were obtained by crossing male and female of heterozygous for C57BL/6N-Dusp1^em1cyagen^ using CRISPR/Cas-mediated genome engineering (Cyagen Biosciences). The Sting exon 3, 4, 5-floxed mice (Sting^flox/flox^, stock#031670, Jackson Lab) and Ksp-cadherin-Cre mice (stock#012237, Jackson Lab) were crossed to generate tubule-specific Sting knockout (Sting-cKO) mice Sting^flox/flox^ Cre. Dusp1 knockout and Sting double knockout mice (Dusp1^−/−^ with Ksp-Cre/Sting^flox/flox^) were generated by crossing Dusp1 knockout mice with Ksp-Cre/Sting^flox/wt^ mice. All mice were bred and maintained under specific pathogen–free conditions at the Experimental Animal Center of Three Gorges University. All mice were age-matched and then randomized into the different groups. The animal research program was approved by the Institutional Review Committee (Ethics Committee) of the Ministry of Health of the People’s Republic of China and the Ethics Committee of Three Gorges University (202205010T2).

### Bilateral/unilateral renal ischemia-reperfusion injury (IRI) model

The bilateral/unilateral renal pedicle was clamped for 28 min with a microaneurysm clamp, as previously mentioned [12]. The renal pedicle was exposed in the sham operation without clamping. For unilateral IRI model, the right kidney of the unilateral left renal ischemia-reperfusion model needs to be removed 24 h before euthanasia. Sp600125 (MedChemExpress, stock in DMSO) diluted in PBS (30 mg/kg) or vehicle were administered intraperitoneally to inhibit JNK activation.

### Cell culture and treatment

The mouse proximal tubular cells (mPTCs) were originally obtained from Sciencell Research Laboratories and cultured in DMEM/F-12 medium supplemented with 10% fetal bovine serum and growth factors. The cells were incubated with H_2_O_2_ at 500 μM for 6 h. Small interference RNAs (siRNAs) were synthesized by Sangon Biotech (Shanghai) Co., Ltd. Small interfering RNAs were transfected by Lipofectamine RNAiMAX reagent (Thermo Fisher Scientific; 13778030), according to the manufacturer’s protocol. A non-silencing siRNA oligonucleotide that does not recognize any known homolog of mammalian genes was used as a negative control. The pcDNA3.1-Dusp1 expressing plasmid was transfected by Lipofectamine 3000 (Thermo Fisher Scientific; L3000001), according to the manufacturer’s protocol. A pcDNA3.1 was used as a negative control. Plasmids were transfected into mouse proximal tubular cells (mPTCs) following incubation for 48 h. Specific silencing or overexpression of the targeted Dusp1 gene was confirmed by western blot analysis.

### Histology

For histologic analysis, kidney tissue was embedded in paraffin and cut into 4 μm thick sections for Hematoxylin and Eosin (H&E), Masson, Periodic Acid-Schiff stain (PAS), immunohistochemistry and immunofluorescence staining. Tubular injury was examined in a blinded manner and scored by the percentage of injured tubules, as follows: 0, no damage; 1, <25%; 2, 25–50%; 3, 50–75%; 4, >75%. At least 10 randomly selected fields per mouse were scored for quantification, and the mean was used as the tubular injury score. The ImageJ tool was used to calculate the collagen volume fraction, that is, the percentage of the blue area (collagen) to the total area of each field. Randomly selected 10 fields of view for each mouse kidney under 400× magnification were scored for quantification. For EM observation, the kidneys were perfused with 1 ml of 10 U/ml heparin, followed by 2 ml fixative containing 100 mM sodium cacodylate, 2 mM calcium chloride, 4 mM magnesium sulfate, 4% paraformaldehyde, and 2.5% glutaraldehyde. Damaged mitochondria were defined by loss of electron density and bridging of cristae in more than 20% of the area of a mitochondrion.

### Immunohistochemical staining

We performed immunohistochemical detection using 5 μm mouse slices. After antigen retrieval and blocking, the primary antibodies for immunohistochemistry staining included DUSP1 (#48625 s, Cell Signaling Technology), anti-COX I (bs-3953R), KIM-1 (#AF1817, R&D System, Minneapolis), and smooth muscle actin (α-SMA) (#A5228, Sigma-Aldrich), which were incubated overnight at 4 °C. After incubation with secondary antibodies, DAB staining or ImmPACT® Vector® Red staining was performed, and the positive staining area was quantified using Image-Pro Plus 6.0 software. Percentage of stained area was calculated using the Image J software. Randomly selected 10 fields of view for each mouse kidney under a 200× magnification were scored for quantification.

### Western blot

Kidney tissues and cells were homogenized and sonicated in RIPA lysis buffer (beyotime, Shanghai, China), which included a protease inhibitor cocktail. The target proteins were separated by SDS/PAGE gel and then transferred to PVDF membranes of 0.22 μm. After blocking with 5% bovine serum albumin (BSA), PVDF membranes were incubated with primary antibodies, including anti-DUSP1 (Abcam, #ab195261), anti-COXI (bs-3953R), anti-p-JNK (Abcam, # ab76572), anti-JNK (Abcam, #ab307802), anti-BAX (Cell Signaling Technology, #2772), anti-cGAS (Cell Signaling Technology, #31659), anti-STING (Cell Signaling Technology, #13647), anti-Cytochrome C (Santa Cruz, sc-13156), and anti-COX IV(Abcam, #ab16056) overnight at 4 °C. HRP-labeled secondary antibodies were employed for membrane incubation for 1 h. Films were scanned and detected with a Bio-Rad calibrated densitometer. The ImageJ software was used for Western blot image analysis.

### Immunofluorescence

The mouse proximal tubular cells (mPTCs) were washed with phosphate-buffered saline (PBS) and fixed with 4% paraformaldehyde for 30 min at room temperature, followed by permeabilization with 0.5% Triton X-100 for 10 min. mPTCs were fixed and permeabilized sequentially by incubating them in acetone and methanol at −20 °C for 20 min. the cells were subjected to overnight incubation with primary antibodies, followed by incubation with secondary antibodies. The primary antibodies used in this study were as follows: anti–Cytochrome C (#sc-13156, Santa Cruz), anti-dsDNA (#ab27156, abcam), anti–cGAS (#31659, Cell Signaling Technology) and anti-Bax (#60267-1-Ig, Proteintech).These secondary antibodies included goat anti-rabbit IgG conjugated with Alexa Fluor 488 or 647, anti-mouse IgG conjugated with Alexa Fluor 488 or 647, or goat anti-rabbit IgG secondary antibody labeled with Alexa Fluor 405 (Jackson Immuno Research). The cell nuclei were stained with 4′,6-diamidino-2-phenylindole (DAPI) at room temperature for 10 min. Cells were stained with 0.1 mM MitoTracker red or green (ThermoFisher Scientific) for 30 min to observe mitochondria. The images were captured using a confocal laser scanning microscope (Leica).

### Isolation of mitochondria

The mitochondrial isolation was performed using the Mitochondria/Cytosol Fractionation Kit (#ab65320, Abcam). Mitochondria were isolated on ice and prepared as mitochondrial and cytosolic fractions for immunoblot analysis.

### Detection of Cytosolic mtDNA by PCR

The mPTCs were lysed in cell lysis buffer and then centrifuged at 700 × *g* for 10 min at 4 °C to remove the cell nuclei and intact cells. The volume of the supernatant was normalized based on the protein concentration. The cell lysate was further centrifuged at 10,000 × *g* for 30 min at 4 °C to separate the cytoplasmic fraction, and then the DNA from the cytoplasmic fraction was isolated. Quantitative PCR was performed to detect mtDNA using primers targeting the mitochondrial cytochrome c oxidase subunit 1 (mtCOI) gene. PCR was also performed to detect nuclear DNA using primers targeting the 18 S ribosomal RNA gene (18 S rDNA). The mtDNA copy number was normalized to the nuclear DNA copy number, and inter-group comparisons were made. The primers for 18 S rDNA were 5′-TGTGTTAGGGGACTGGTGGACA-3′ (forward) and 5′-CATCACCCACTTACCCCCAAAA-3′ (reverse). The primers for mtCOX2 were 5′-ATAACCGAGTCGTTCTGCCAAT-3′ (forward) and 5′-TTTCAGAGCATTGGCCATAGAA-3′ (reverse).

### Measurement of mitochondrial ROS (mtROS) and mitochondrial transmembrane potential

The cells were incubated with MitoSox Red fluorescent dye (5 μM, Thermo Fisher, USA) at 37 °C for 30 min. The cells were then washed three times with warm PBS buffer. Finally, the cells were observed using a Leica confocal microscope. JC-1 (C2006; Beyotime) was used to measure the mitochondrial membrane potential (MMP). The experiment was performed as described previously in the literature [12] and according to the manufacturer’s instructions.

### Mitochondrial respiration measurement

The mitochondrial oxygen consumption rate (OCR) was analyzed using a Seahorse XFe24 Analyzer (Agilent Technologies, Inc., North Billerica, MA, USA) to measure mitochondrial respiration. Briefly, cells were seeded at a density of 2.0 × 10 ^ 5 cells per well in a 24-well XFe24 cell culture microplate and treated according to the designed method. In the respiration assay, cells were exposed to H_2_O_2_ for 6 h, and OCR was measured every 3 min for at least 90 min. Firstly, OCR was quantified under basal conditions (20 mM glucose), with 1 μM oligomycin (an ATP synthase inhibitor) and 0.125 μM FCCP (a mitochondrial respiratory uncoupler), and finally with 1 μM rotenone/antimycin A (complex I and III inhibitors). OCR was automatically calculated using the Seahorse XF-24 software.

### ATP measurement

The ATP concentration in mPTCs was measured using an ATP fluorescence assay kit (S0026; Beyotime) according to the manufacturer’s protocol.

### Coimmunoprecipitation

Endogenous co-IP was carried out to analyze the interaction between DUSP1 and JNK in mPTC cells. Briefly, cells were lysed in mild cell lysis buffer (P0013; Beyotime). After centrifugation at 14,000 g for 10 min, the supernatant was incubated overnight at 4 °C with control IgG or anti-DUSP1 (Cell Signaling Technology, #48625 s), and then precipitated with protein A/G PLUS agarose (Santa Cruz). The immunoprecipitated complexes were washed and eluted for immunoblot analysis.

### Statistical analysis

All quantitative data are presented as mean ± standard error of the mean (SEM). Results were analyzed using Prism 9.0 software (GraphPad, San Diego, CA). Normal distribution and homogeneity of variance were tested using Shapiro–Wilk test and Bartlett s test, respectively. For data that passed both normality and equality of variance, comparisons between two groups were performed using the unpaired 2-tailed Student’s *t* test, and multiple comparisons were analyzed using the analysis of variance (ANOVA) followed by Tukey’s post-hoc test. If not, the non-parametric Kruskal–Wallis test was applied, followed by Dunn’s test. *P* < 0.05 was considered as the level of significance.

## Results

### Dusp1 expression is increased in transplanted kidneys from deceased donors as well as in mouse kidneys following IRI

**Fig. 1:**
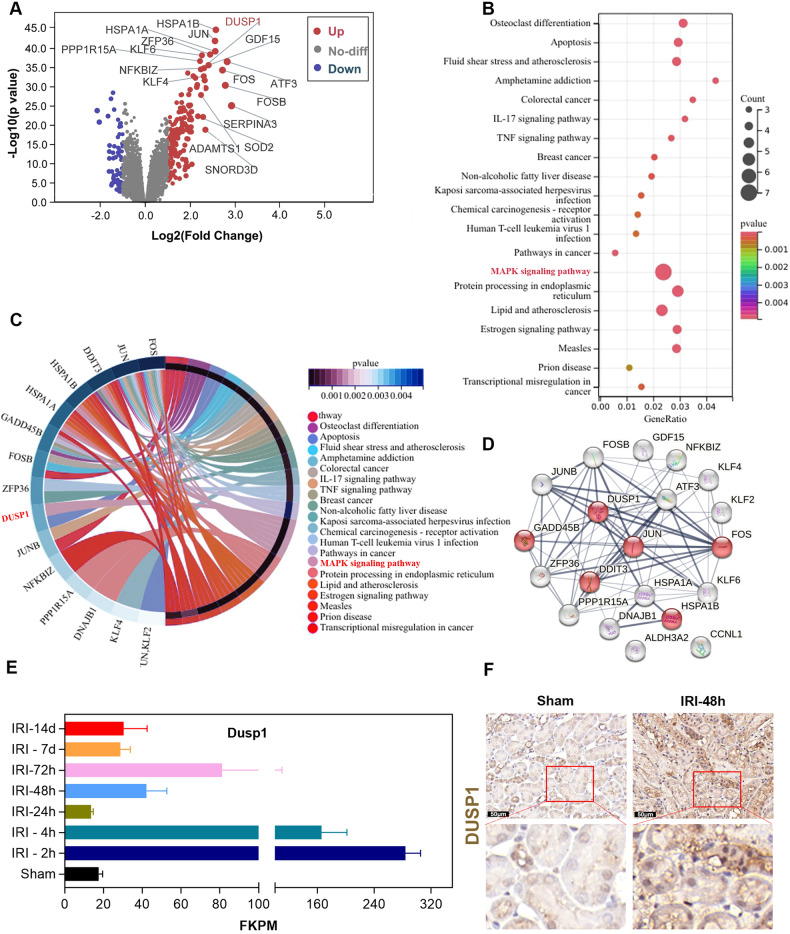
Dusp1 expression is upregulated after renal ischemia-reperfusion in humans and mice. **A** Volcano of DEGs by screening the GSE43974 dataset. The up-regulated DEGs(red) were labeled. **B** KEGG enrichment pathways for top 20 DEGs. **C** Chord plots show KEGG-enriched items of DEGs. **D** Top 20 DEGs encoded protein interaction network analysis. **E** Temporal FPKM expression trends based on Dusp1 during renal IRI. **F** Representative immunohistochemical staining images demonstrating kidney DUSP1 expression in sham-operated and 48 h after IRI.

Transplanted kidneys from deceased donors inevitably suffer IRI, which may lead to AKI and affect graft survival. To identify potential targets affecting the prognosis of kidney transplantation, we analyzed the whole genome microarray transcriptional profile of human kidney biopsy samples (GSE43974) from a total of 554 kidney biopsy tissues collected from living and deceased donors at the time of donation, after cold ischemia, and after reperfusion [27]. The transcriptional levels of GDF15, ATF3, JUN, FOS, KLF6, DUSP1, and others were up-regulated, of which GDF15, ATF3, JUN, FOS, and KLF6 have been identified in renal IRI, while the role of DUSP1 remains unknown (Fig. 1A). Our results showed that compared to living donor kidney transplants, various pathways related to cell apoptosis, IL-17 signaling, MAPK signaling, and TNF signaling were activated in the kidney tissue of deceased brain death donors (DBD) as shown in Fig. 1B. Furthermore, the chord diagram and STRING protein-protein interaction analysis demonstrated that among the top 20 differential genes, which include DUSP1, JUN, GADD458, DDIT3, HSPA18, and FOS6, all are associated with the MAPK signaling pathway. (Fig. 1C, D). Interestingly, analysis of transcriptome data (GSE98622) of mouse kidney tissues at different time points also showed the increased expression of DUSP1 after IRI (Fig. 1D). Additionally, we confirmed the significant up-regulation of DUSP1 levels in renal tubular cells of AKI mice kidney samples through immunohistochemistry (Fig. 1E).

### The absence of Dusp1 exacerbates ischemic AKI in mice

**Fig. 2:**
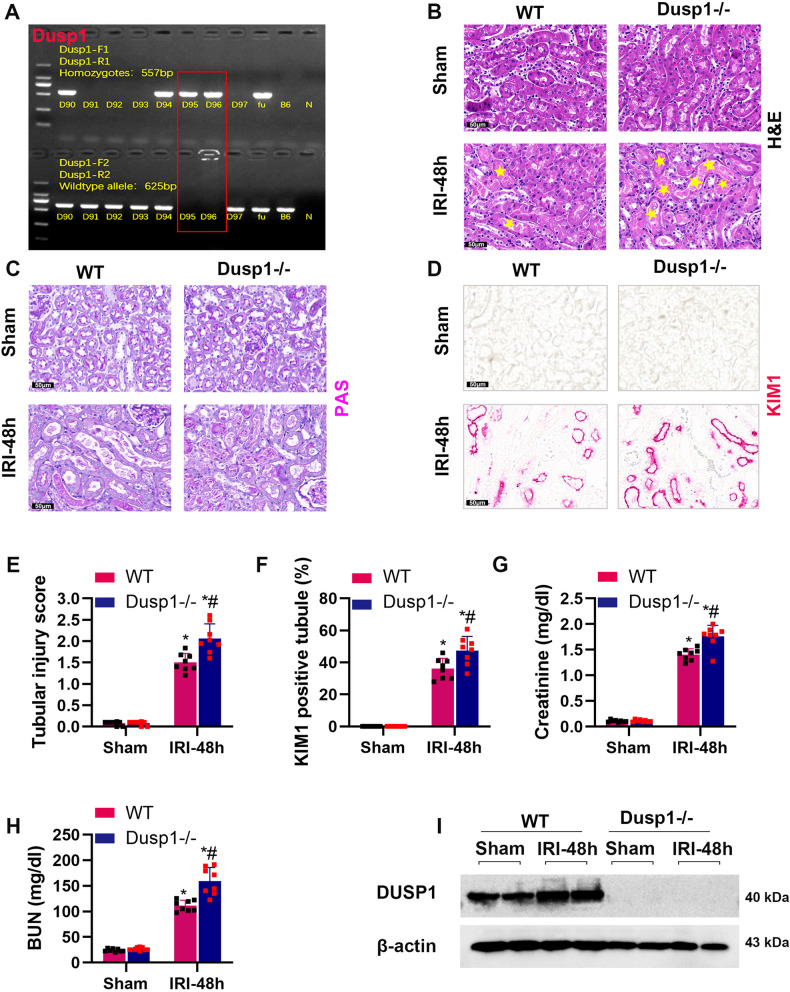
Protective effect of Dusp1 on kidney after 48 h of reperfusion followed by ischemia. **A** Identification of Dusp1 knockout mice. **B** Representative graphs of kidney sections with H&E staining (400×). **C** Representative periodic acid-Schiff (PAS)-stained histological sections are presented (400×). **D** Representative images of KIM1 immunohistochemistry (400×). **E** Quantification of the tubular damage score. **F** Quantitative analysis of Kim-1 positive tubules. **G** Serum creatinine measurement. **H** Blood urea nitrogen measurement. **I** Representative Western blot and quantitative data showing the Dusp1 protein abundance in sham or IRI- treated kidneys from wild-type (WT) and Dusp1^−/−^ mice. *p < 0.05 versus respective sham-operated group (*n* = 8). ^#^*p* < 0.05 versus WT IRI-48h group (*n* = 8).

To evaluate the potential role of DUSP1 in IRI-induced AKI, we generated Dusp1 knockout mice (Fig. 2A) and established AKI models with Dusp1 knockout (Dusp1^−/−^) and wild-type (WT) mice (bilateral ischemia for 28 min followed by reperfusion for 48 h). Under non-ischemic conditions, Dusp1 knockout did not affect the renal structure and function. However, after ischemic intervention, Dusp1^−/−^ mice exhibited significantly increased levels of renal tubular pathological injury (Fig. 2B, C), expression of kidney injury molecule 1 (KIM-1) (Fig. 2D, F), kidney injury score (Fig. 2E), creatinine (Fig. 2G) and blood urea nitrogen levels (Fig. 2H) compared to WT mice. Western blot results validated the *absence of DUSP1 protein* in Dusp1-deficient mice (Fig. 2I). Taken together, these data suggested that the absence of Dusp1 was associated with renal dysfunction and tubular injury in renal IRI.

### Deletion of Dusp1 exacerbates post-ischemic renal fibrosis and dysfunction

**Fig. 3:**
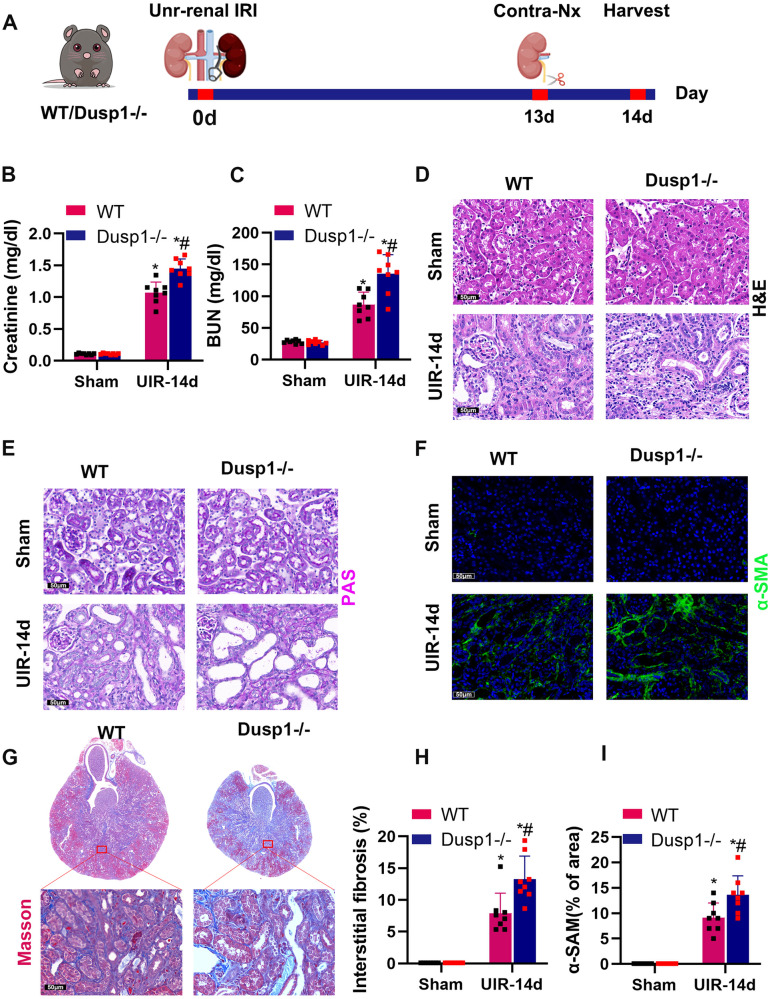
Dusp1 deficiency promoted post-ischemic renal fibrosis. **A** Schematic for renal IRI model using WT/Dusp1^−/−^ male mice (details are in Methods). **B** Serum creatinine measurement. **C** Blood urea nitrogen measurement. **D** Representative graphs of kidney sections with H&E staining (400×). **E** Representative periodic acid-Schiff (PAS)-stained histological sections are presented (400×). **F** Representative graphs of α-SMA immunofluorescence staining (400×). **G**, **H** Representative graphs of Masson staining from WT UIR-14d and Dusp1^−/−^ UIR-14d groups mice, and semi-quantification analysis. **I** Quantification of the α-SMA Immunofluorescence. *p < 0.05 versus respective sham-operated group (*n* = 5). ^#^*p* < 0.05 versus WT UIR-14d group (*n* = 5).

To further elucidate the long-term impact of Dusp1 knockout on renal IRI, we established a model of post-ischemic fibrosis. Unilateral (left) renal ischemia for 28 min followed by reperfusion was performed to induce renal fibrosis, and on postoperative day 13, the right kidney was removed and blood and left kidney tissue were collected for analysis on day 14 (Fig. 3A). Creatinine and blood urea nitrogen levels indicated that Dusp1^−/−^ mice had significantly worse renal functional recovery after IRI (Fig. 3B, C). Hematoxylin and eosin (H&E) and periodic acid-Schiff (PAS) staining revealed brush border loss, tubular dilation, and inflammatory infiltration in post-ischemic kidneys, which were exacerbated in dusp1 knockout mice (Fig. 3D, E). Immunostaining for α-smooth muscle actin (α-SMA) and Masson’s trichrome staining showed shrunken kidneys, tubular atrophy, and collagen deposition at day 14 after renal IRI. Compared with WT mice, Dusp1^−/−^ mice significantly exacerbated renal atrophy and fibrosis 14 days after IRI (Fig. 3F–I). These results indicated that Dusp1 contributed to the progression of CKD and fibrosis.

### Deletion of Dusp1 exacerbates mitochondrial damages in tubular epithelial cells induced by ischemia-reperfusion injury (IRI)

**Fig. 4:**
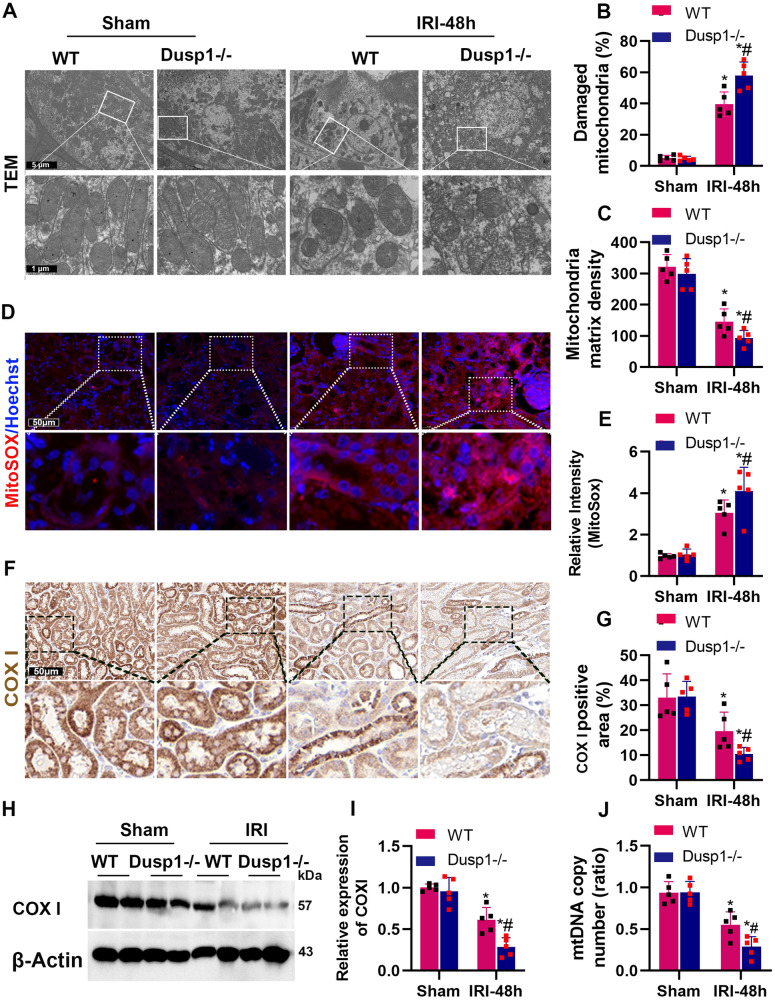
Dusp1 deficiency worsened ischemia-induced proximal tubular mitochondrial injury in vivo. **A** Representative TEM images of mouse kidney tissues. **B** Quantitative analysis of damaged mitochondria from electron microscopy images. **C** Quantitative analysis of mitochondrial matrix density. **D** Representative images for MitoSOX staining (**E**) Quantitative analysis of MitoSOX staining. **F** Representative images and (**G**) Quantitative analysis of COX I immunohistochemical (IHC) staining. **H** Representative Western blot of COX I (**I**) Quantitative data showing the COX I protein abundance in sham or IRI- treated kidneys from wild-type (WT) and Dusp1^−/−^ mice. **J** Measurement of mtDNA copy number levels in the kidneys on 48 h after IRI-AKI. *p < 0.05 versus respective sham-operated group (*n* = 5). ^#^*p* < 0.05 versus WT IRI-48h group (*n* = 5).

Mitochondria dysfunction plays a crucial role in the development of AKI and AKI-CKD, and DUSP1 has been reported to have a significant impact on mitochondrial function [28]. Transmission electron microscopy (TEM) results showed mitochondrial damage, swelling of cristae, and dense matrix in some renal tubular cells of IRI mice. Compared to WT, Dusp1 knockout mice exhibited worsened mitochondrial morphological damage and a decrease in mitochondrial matrix (Fig. 4A–C). Next, MitoSOX and Cytochrome c oxidase subunit I (COX I) immunohistochemistry were used to analyze mitochondrial ROS production and mitochondrial content. IRI challenge significantly increased ROS levels in renal tissue (Fig. 4D, E) and decreased COX I (Fig. 4F, G), while Dusp1 deficiency worsened these changes. Western blot results verified that Dusp1-deficient mice exhibited a more significant reduction in cytochrome C induced by IRI. (Fig. 4H, I). Meanwhile, IRI induced a decreased level of mitochondrial DNA copy number and Dusp1 deletion worsened the decreasing trend (Fig. 4J). Collectively, these results demonstrate that the absence of Dusp1 exacerbates mitochondrial damage in renal tubular epithelial cells caused by IRI.

### The absence of Dusp1 exacerbates H_2_O_2_-induced mitochondrial damages and mtDNA release

**Fig. 5:**
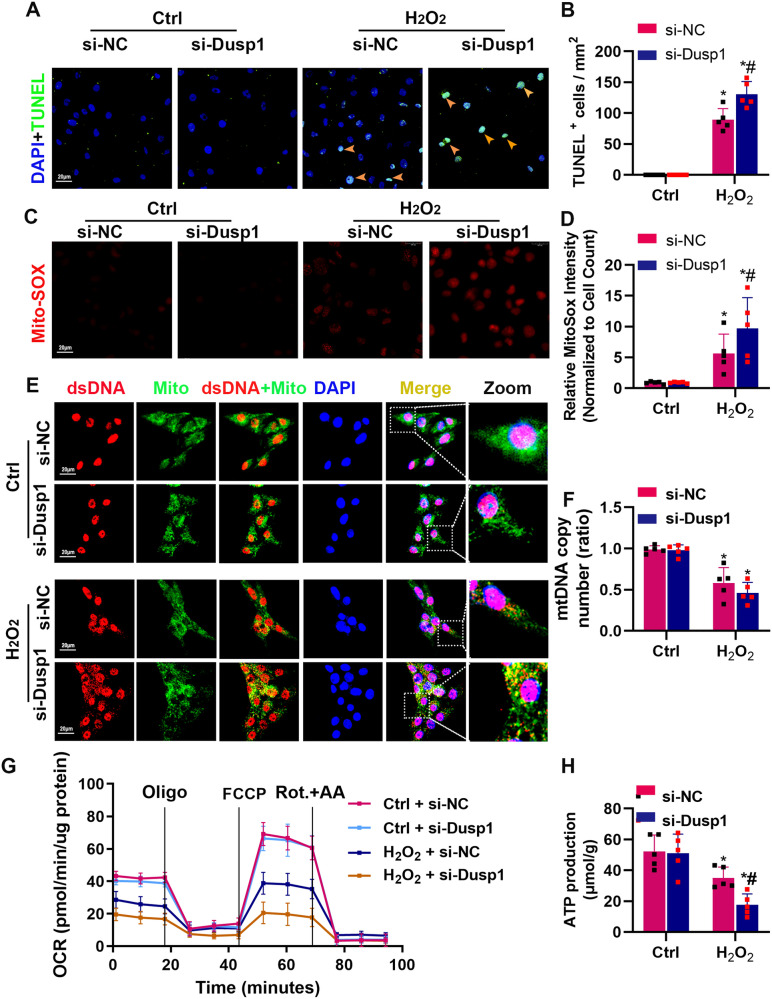
Dusp1 knockdown exacerbated mitochondrial dysfunction and mitochondrial DNA release in mouse proximal tubular cells (mPTCs) induced by H_2_O_2_. **A** Representative photographs of terminal deoxynucleotidyl transferase–mediated dUTP nick end-labeling (TUNEL) staining (green) and (**B**) Quantification of TUNEL-positive staining in the tubular cells per mm^2^ in the mPTCs. **C** Representative images of MitoSOX staining (**D**) Quantitative analysis of MitoSOX staining. **E** Representative immunofluorescence images of mPTCs stained with anti-Mito (green) and anti–double-strained DNA (dsDNA; red) antibodies and 4,6-diamidino-2-phenylindole (DAPI; blue). **F** Copy number levels of mtDNA in mPTCs with si-Dusp1 or si-NC in the presence or absence of H_2_O_2_ challenge. **G** Measurement of oxygen consumption rate (OCR) using anXF 24 Extracellular Flux Analyzer (Seahorse Bioscience). *n* = 5 in each group. **H** Semiquantitative analysis of ATP production in the different groups. *P < 0.05 versus respective control, ^#^*P* < 0.05 versus pc-DNA3.1 with H_2_O_2_. (*n* = 5).

To mimic IRI in vivo, H_2_O_2_ was used to treat renal tubular cells. We examined the regulatory role of Dusp1 in H_2_O_2-_induced tubular injury. We found that Dusp1 siRNA exacerbated H_2_O_2_-induced cell death (Fig. 5A, B) and mitochondrial ROS production (Fig. 5C, D), while Dusp1 overexpression preserved mitochondrial membrane potential (Supplementary Fig. 1). H_2_O_2_ impaired the oxygen consumption rate (OCR) in renal tubular epithelial cells, including ATP production and maximum respiration, and this effect was exacerbated by Dusp1 deficiency (Fig. 5G, H). Hydrogen peroxide led to mitochondrial damage and permeability changes, which released mitochondrial DNA (mtDNA), a newly discovered important factor in the conversion of AKI to CKD. Dusp1 knockdown did not affect total mtDNA copy number in cultured cells (Fig. 5F). To visualize mtDNA distribution, mtDNA release was assessed using dsDNA immunofluorescence. The results demonstrated that H2O2-induced dsDNA signals distributed around the mitochondria, and this phenomenon was more pronounced in cells with reduced DUSP1 expression (Fig. 5E). These data suggest that the deficiency of Dusp1 aggravates H_2_O_2_-induced mitochondrial damage and mtDNA release in renal tubular epithelial cells.

### The absence of Dusp1 activates BAX and promotes cGAS/STING-mediated inflammatory signaling

**Fig. 6:**
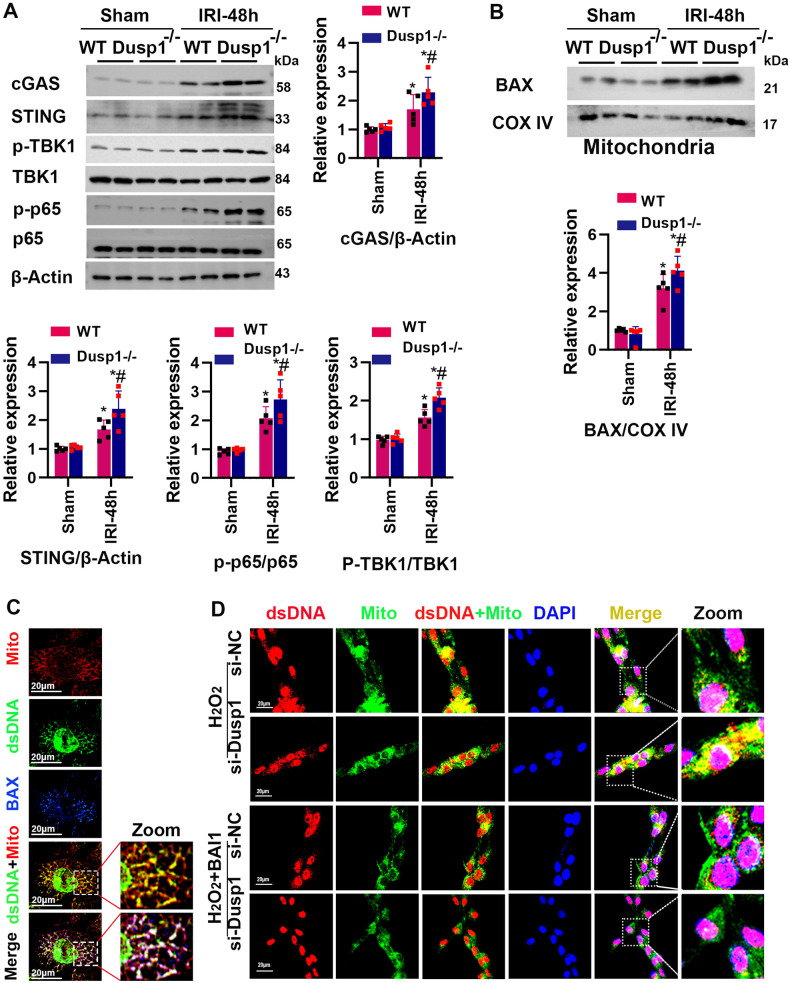
The deficiency of Dusp1 mediates cGAS activation and mtDNA release, which can be inhibited by BAX inhibitor (BAI1). **A** Representative Western blot and quantitative data showing cGAS, STING, p-TBK1, TBK1 p-p65, and p65 expression in sham or IRI- treated kidneys from wild-type (WT) and Dusp1^−/−^ mice. *p < 0.05 versus respective sham-operated group (*n* = 5). ^#^*p* < 0.05 versus WT IRI-48h group (*n* = 5). **B** Representative Western blot and quantitative data showing Bax in mitochondria (*n* = 5). **C** Immunofluorescence of H_2_O_2_-treated (500 μM of H_2_O_2_ for 6 h) cells labeled against dsDNA (green), BAX (blue), and mitochondria (Mito; red). **D** Immunofluorescence staining images of the release of mtDNA into the cytoplasm. *p < 0.05 versus the respective control group (*n* = 5). ^#^*p* < 0.05 versus si-NC H_2_O_2_ group (*n* = 5).

IRI-induced tubular cell injury and mitochondrial damage, particularly the increased permeability of mitochondrial membranes, can lead to the release of mtDNA, which has been shown to induce the cGAS/STING pathway and affect the AKI outcome [18]. By comparing wild-type and Dusp1^−/−^ knockout mice, we found the overactivation of STING pathway in response to renal IRI in Dusp1^−/−^ mice including increased expression of cGAS, Sting, p-TBK1 and p-p65 (Fig. 6A). Based on previous reports that mtDNA is released from mitochondria through BAX pores [29], we examined the mitochondria BAX in isolating mitochondria from renal tissue. Dusp1 gene deletion promoted the increase of BAX translocation to mitochondria in response to IRI (Fig. 6B). We further evaluated the contribution of BAX to mtDNA leakage into the cytoplasm by triple-fluorescence microscopy analysis of dsDNA, mitochondria, and BAX in H_2_O_2_-treated mouse renal tubular epithelial cells. In H_2_O_2_-treated mouse renal tubular epithelial cells, cytoplasmic mtDNA appeared to co-localize with BAX, near the mitochondria (Fig. 6C). To further confirm whether Dusp1 exerts its function through BAX activation, BAX inhibitor (BAI1) was used in vitro to evaluate the mtDNA release in each group. Knockdown of Dusp1 by siRNA-Dusp1 increased the release of dsDNA and BAI1 significantly attenuated the mtDNA leakage caused by Dusp1 knockdown (Fig. 6D). Overall, these data suggested that Dusp1 deficiency further activates BAX after H_2_O_2_ treatment, exacerbates mtDNA leakage, and activates the cGAS-STING pathway.

### Depletion of DUSP1 activates BAX by increasing JNK phosphorylation

**Fig. 7:**
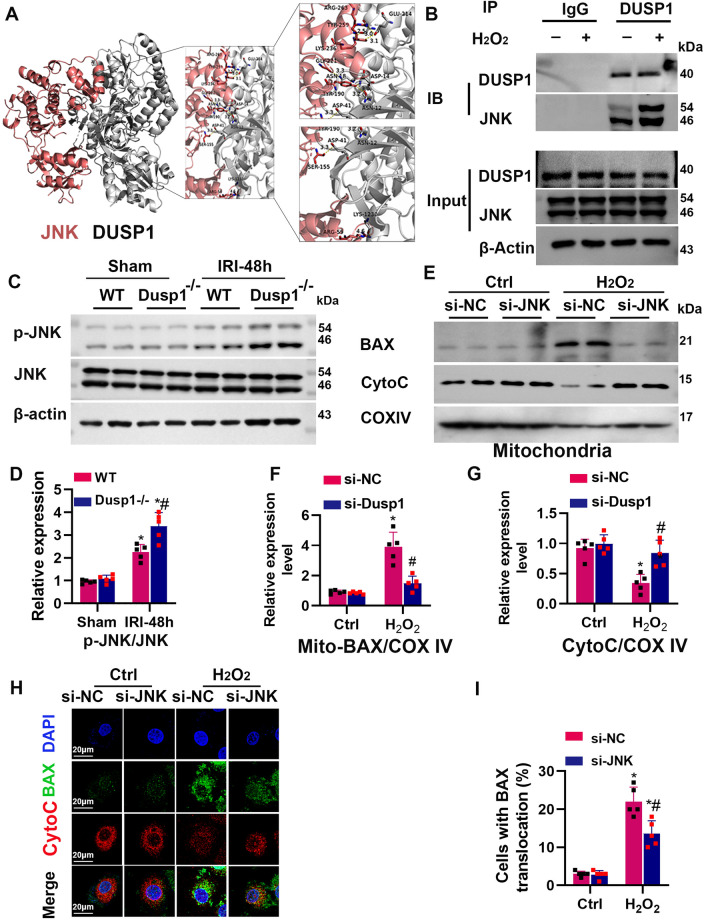
DUSP1 deficiency activates BAX via increasing JNK phosphorylation. **A** Structure-based protein interaction interface analysis between DUSP1 and JNK. Images represent the predicted DUSP1-JNK complex structure, where interaction hotspot residues are labeled. **B** Coimmunoprecipitation of DUSP1 and JNK. Cell lysates isolated from normal cultured or H_2_O_2_-treated cells were immunoprecipitated by anti-DUSP1 antibody (rabbit anti-IgG antibody as a negative control) and then immunoblot analyzed anti-DUSP1 and anti-JNK antibodies (*n* = 3). IB immunoblot, IP immunoprecipitation. **C**, **D** Representative Western blot and quantitative data showing the JNK protein abundance in sham or IRI- treated kidneys from wild-type (WT) and Dusp1^−/−^ mice. *p < 0.05 versus respective sham-operated group (*n* = 5). ^#^*p* < 0.05 versus WT IRI-48h group (*n* = 5). **E**–**G** Representative Western blot and quantitative data showing the effects of JNK gene silencing on BAX protein abundance from mitochondria (Mito) and cytoplasm (Cyto) in mouse proximal tubular cells (mPTCs) with or without H_2_O_2_ treatment. *p < 0.05 versus the respective control group (*n* = 5). ^#^*p* < 0.05 versus si-NC with H_2_O_2_ group (*n* = 5). **H** Representative immunofluorescence images of mPTCs stained with anti-BAX (green) and Cytochrome c (Cytoc) (red) antibodies and DAPI (blue). **I** Quantification of Cells with BAX translocation *p < 0.05 the versus respective control group (*n* = 5). ^#^*p* < 0.05 versus si-NC with H_2_O_2_ group (*n* = 5).

To identify potential interacting partners of DUSP1, we downloaded the three-dimensional protein structure files of JNK and Dusp1 from the RCSB database (https://www.rcsb.org/). We performed three-dimensional and two-dimensional force analysis and visualization using Discovery Studio and Ligplus software. The results showed that the binding energy between JNK and Dusp1 was −276.25 kcal/mol. In the two-dimensional image, green dotted lines represent hydrogen bonds, while red dotted lines represent hydrophobic interactions (Supplementary Fig. 2). Typically, if the binding energy between the ligand and target protein is more negative, the binding is more stable. In the three-dimensional image, the red ribbon-like structure represents the JNK protein, while the silver-white ribbon-like structure represents the Dusp1 protein (Fig. 7A). CO-immunoprecipitation also showed that Dusp1 could directly bind with JNK (Fig. 7B). Moreover, Dusp1 gene deficiency further upregulated p-JNK levels after renal IRI (Fig. 7C, D), suggesting that Dusp1 may directly protect against JNK over phosphorylation in renal IRI. To test whether JNK activation affects the distribution of BAX after H2O2 treatment, we measured the distribution of BAX in mPTCs mitochondria and cytoplasm. The results showed that JNK silencing significantly increased the content of mitochondrial BAX (Fig. 7E–G). Furthermore, co-staining of BAX (green fluorescence) and cytochrome C (red fluorescence) showed that JNK silencing prevented the mitochondrial translocation of BAX (Fig. 7H, I). These suggested that JNK may be a potential intermediate protein that mediates the regulatory effect of DUSP1 on BAX activation.

### Inhibition of JNK can alleviate exacerbation of ischemia-reperfusion injury caused by DUSP1 deficiency

**Fig. 8:**
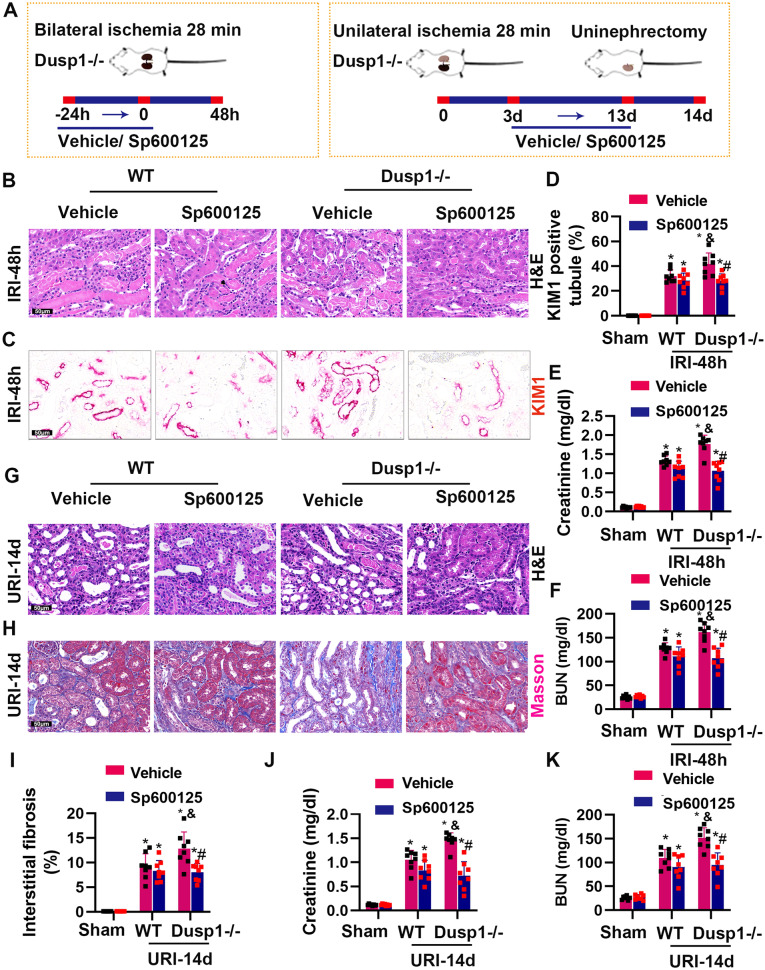
Pharmacological inhibition of the JNK attenuates tubular injury and renal fibrosis in DUSP1-/- mice in AKI and CKD models. Vehicle or JNK inhibitor, Sp600125, was administered 24 h before I/R injection and repeated every 24 h. **A** Schematic for renal IRI model with Dusp1^−/−^ male mice (details are in Methods). **B** Representative graphs of kidney sections with H&E staining from respective IRI-48h groups mice (400×). **C** Representative images of KIM-1 immunohistochemistry (400×). **D** Quantitative analysis of KIM-1 positive tubules. **E** Serum creatinine measurement from respective IRI-48h groups mice. **F** Blood urea nitrogen measurement from respective IRI-48h groups mice. **G** Representative graphs of kidney sections with H&E staining from respective UIR-14d groups mice (400×). **H** Representative graphs of Masson staining from respective UIR-14d groups mice, and (**I**) semi-quantification analysis. **J** Serum creatinine measurement from respective UIR-14d groups mice. **K** Blood urea nitrogen measurement from respective UIR-14d groups mice. *p < 0.05 versus respective sham-operated group. ^#^*p* < 0.05 versus vehicle IRI-48h group or vehicle UIR-14d group. ^&^*p* < 0.05 versus Dusp1^+/+^ IRI-48h group (*n* = 8).

Next, we investigated whether JNK inhibitor (Sp600125) can alleviate AKI and CKD in Dusp1-deficient mice. AKI model was established by bilateral ischemia for 28 min followed by reperfusion for 48 h, and Sp600125 was administered twice via intraperitoneal injection 24 h and immediately before surgery (Fig. 8A). In the AKI model, Sp600125 offered moderate protection to WT mice, however, it significantly attenuated renal pathological injuries, renal dysfunction and KIM1 expression in Dusp1-knockout mice (Fig. 8B–F). Unilateral ischemia for 28 min followed by reperfusion was performed to establish renal fibrosis model, and Sp600125 was injected intraperitoneally daily from postoperative day 3 to day 13. Right kidney was removed on day 13 and blood and left kidney tissue were collected for analysis on day 14 (Fig. 8A). In Dusp1^−/−^ mice, Sp600125 treatment ameliorated renal function, renal pathology, renal atrophy, and collagen deposition compared to the vehicle group (Fig. 8G–K). These data demonstrated that DUSP1 deficiency leaded to excessive activation of JNK and that inhibition of JNK can alleviate exacerbation of IRI caused by DUSP1 deficiency.

### Sting deficiency alleviates the adverse effects of Dusp1 deficiency

**Fig. 9:**
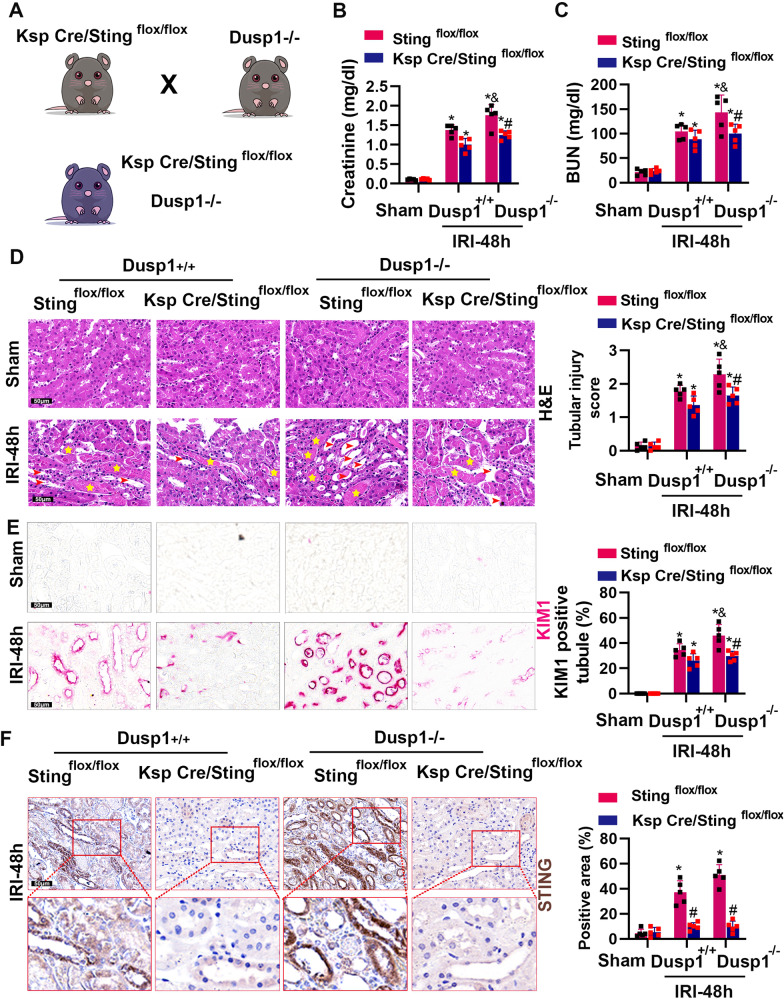
Genetic deletion of Sting attenuates the deteriorated influence of Dusp1 loss in IRI-treated kidneys. **A** Experimental scheme for the generation Dusp1^−/−^ with Ksp-Cre/Sting^flox/flox^ mice. **B** Serum creatinine measurement. **C** Blood urea nitrogen measurement. **D** Representative graphs of kidney sections with H&E staining (400×) and quantification of the tubular damage score. **E** Representative images of KIM-1 immunohistochemistry (400×) and quantitative analysis. **F** Representative immunohistochemical staining images and quantitative analysis demonstrating Sting expression in 48 h after IRI (400×). *p < 0.05 versus respective sham-operated group. ^#^*p* < 0.05 versus respective Sting^flox/flox^ IRI-48h group; ^&^*p* < 0.05 versus Dusp1^+/+^ IRI-48h group (*n* = 5).

To further investigate whether cGAS-STING pathway mediated the observed effect of Dusp1 in AKI mice, we generated mice with double knockout of Dusp1 and Sting by crossing Dusp1^−/−^ mice with Ksp-Cre/Sting ^flox/flox^ mice and subjected them to IRI for 48 h (Fig. 9A). Immunohistochemical staining for STING showed that there was a significant upregulation of STING in the kidneys of Sting ^flox/flox^ mice while there was less expression in the kidneys of Ksp-Cre/Sting ^flox/flox^ mice after IRI (Fig. 9F). We found that the double deletion of Dusp1 and Sting did not affect the kidney structure and function in non-ischemic conditions. Importantly, all the changes, including KIM1, renal function impairment and kidney morphological changes were blunted in double-knockout mice compared to Dusp1^−/−^ knockout mice after ischemic challenge (Fig. 9B–E). These data above suggested that tubular Sting pathway activation mediated the adverse effects of Dusp1 deficiency in AKI.

## Discussion

In this study, we revealed that the activation of DUSP1 is a defensive protective mechanism against renal IRI. Specifically, in renal IRI model, our findings show that: (1) DUSP1 expression is significantly upregulated in human DBD kidney tissue and mouse AKI kidney tissue; (2) Dusp1 knockout mice exacerbates IRI-induced renal injury, kidney fibrosis, and tubular epithelial cell mitochondrial damage; (3) Silencing of DUSP1 aggravates H_2_O_2_-induced tubular injury, mitochondrial damage, and mtDNA release; (4) loss of DUSP1 increases JNK phosphorylation, activates BAX, and promotes cGAS/Sting-mediated inflammatory signaling; (5) the deficiency of STING or inhibition of JNK can significantly reduce the adverse effects caused by DUSP1 deficiency.

The dual-specificity phosphatase (DUSP) protein family consists of 11 members and 3 categories (Category I: DUSP 1, 2, 4, and 5 in the nucleus; Category II: DUSP 6, 7, and 9 are found in the cytoplasm; Category III: DUSP 8, 10, and 16 are present in both nucleus and cytoplasm) [30]. As an enzyme that can remove phosphate groups from tyrosine and serine residues, DUSP plays a crucial role in cellular activities, including mitophagy [31], mitochondrial fission [32], and mitochondrial metabolism [25]. In this study, our data showed that Dusp1 expression was elevated in brain death donor (DBD) kidney samples and AKI mouse kidneys (Fig. 1). Dusp1 gene deletion significantly exacerbated renal function deterioration, renal tubular injury (Fig. 2), renal fibrosis (Fig. 3), mitochondrial damage (Fig. 4), and the production of inflammatory cytokines induced by IRI. Furthermore, in cultured renal tubular epithelial cells, Dusp1 knockdown exacerbated the release of mitochondrial DNA, accumulation of mitochondrial reactive oxygen species, and reduction of ATP induced by H_2_O_2_ (Fig. 5). These findings are consistent with previous research suggesting that DUSP1 effectively improves mitochondrial damage and dysfunction [25]. Overall, DUSP1 in renal tissue may be a potential target for reducing inflammation and kidney injury [33].

Under conditions of cellular stress and mitochondrial dysfunction, mitochondrial DNA (mtDNA) can be released into the cytoplasm or extracellular space, which not only enhances antimicrobial innate immunity during infection but also potentially drives detrimental inflammatory responses during disease processes [34, 35]. In AKI, mitochondria dysfunction is found to trigger an innate immune response by releasing mitochondrial DNA (mtDNA) outside the mitochondria, which promotes renal damages [18, 36]. Detection of circulating free mtDNA is even considered a potential biomarker for assessing tissue damage [37]. In addition, cytoplasmic mislocalization of mtDNA can induce fibrotic changes in mouse renal tubular epithelial cells [19]. In this study, we reported that the loss of DUSP1 exacerbates mtDNA release into the cytoplasm (Fig. 5), while overexpression of DUSP1 significantly alleviates the reduction of mitochondrial membrane potential (Supplementary Fig. 2), indicating a critical role for DUSP1 in mitochondrial dysfunction and the abnormal leakage of mtDNA. These findings suggested that the role of DUSP1 in regulating mitochondrial function is more diverse than previously understood.

Leaked mtDNA can act as an endogenous ligand for cGAS, thus inducing an innate immune response through the STING pathway [18, 19] We found that Dusp1 deletion further activates the cGAS-STING pathway, accompanied by aggravated renal injury, in an ischemic AKI mouse model (Fig. 6). However, compared to DUSP1 knockout mice, the kidney injury was significantly alleviated in STING and DUSP1 double-knockout mice (Fig. 9). In addition to AKI, chronic cellular stress induced by oxidative stress and mitochondria dysregulation has been found to activate the cGAS-sting pathway, which is associated with progress of CKD [19, 38]. Consistently, the fibrotic changes were alleviated in the Sting and Dusp1 double-knockout mice compared with Dusp1 knockout mice. Therefore, the regulation of mitochondrial damage by DUSP1 affects the activation of the cGAS-STING pathway, providing a new explanation for preventing disease progression in AKI and CKD models.

In order to reveal the molecular mechanism by which DUSP1 loss promoted mtDNA release, we focused on the phosphorylation status of JNK and the mitochondrial translocation of BAX. DUSP1 could dephosphorylate serine/threonine and tyrosine residues of its substrates, especially JNK, to exert its biological function [39–41]. Similarly, in this study, JNK was identified as an interacting partner of DUSP1 in proximal tubular cells by analyzing protein interaction (Fig. 7) and we found that DUSP1 was a key regulator for phosphorylation status of JNK in AKI model (Fig. 7). It has been reported that activated JNK can promote the translocation of BAX to mitochondria [42, 43] through phosphorylation of the 14-3-3 protein [44] and the formation of BAX/BAK pores in the mitochondrial outer membrane was closely related to mtDNA release [29, 45]. Immunofluorescence indicated that cytoplasmic mtDNA and BAX co-localized on the surface of mitochondria in H_2_O_2_-treated renal tubular epithelial cells (Fig. 6C). The BAX inhibitor (BAI1) effectively inhibits mtDNA leakage induced by DUSP1 deficiency under H_2_O_2_ stimulation (Fig. 6). Interestingly, we observed that JNK silencing significantly attenuated the mtDNA release caused by DUSP1 deficiency (Fig. 7), and that treatment of the JNK inhibitor SP600125 reversed the adverse effects on the kidney caused by DUSP1 deficiency in mice (Fig. 8). These data suggested that the loss of DUSP1 led to the overactivation of JNK, causing BAX translocation to mitochondria and mtDNA release under the AKI model.

However, there are still some limitations and unresolved issues. Firstly, the use of systemic knockout mice in the experiments does not exclude the possibility that the lack of DUSP1 in other types of cells, such as immune cells, may also lead to activation of the cGAS-sting pathway. Secondly, previous studies have reported that DUSP1 can alleviate inflammation by improving mitochondrial engulfment and metabolism [25], and we cannot rule out the possibility that the beneficial effect of DUSP1 in AKI-CKD may partly come from the regulation of mitochondrial engulfment and metabolism. In addition, in a previous study on myocardial IRI, DUSP1 was downregulated after acute cardiac IR injury compared to the control group [46]. The exact reason for the inconsistency between the two findings is unclear and may be due to differences in the distribution of DUSP1 in different organs and tissues, animal models, treatment methods, and the setting of differential thresholds. Finally, the precise mechanism by which mtDNA is released from mitochondria through BAX remains unclear.

In summary, we have reported DUSP1 as a defensive protective mechanism to alleviate the process of AKI to CKD transition. DUSP1 inhibits the release of mtDNA through the BAX pore in the mitochondrial outer membrane by dephosphorylating JNK, thereby suppressing the cGAS-STING signaling pathway and subsequent renal injury. Therapeutic strategies targeting DUSP1 and this pathway may offer therapeutic implications for ischemic kidney disease and subsequent renal fibrosis.

### Reporting summary

Further information on research design is available in the Nature Research Reporting Summary linked to this article.

### Supplementary information

Supplementary materials

Authorship Change Approval

Reporting Summary

Editing Certificate

## Data Availability

The data that support the findings of this study are available from the corresponding author upon reasonable request.

## References

[1] Hoste EAJ, Kellum JA, Selby NM, Zarbock A, Palevsky PM, Bagshaw SM (2018). Global epidemiology and outcomes of acute kidney injury. Nat Rev Nephrol.

[2] James MT, Bhatt M, Pannu N, Tonelli M (2020). Long-term outcomes of acute kidney injury and strategies for improved care. Nat Rev Nephrol.

[3] Yang L, Xing G, Wang L, Wu Y, Li S, Xu G (2015). Acute kidney injury in China: a cross-sectional survey. Lancet.

[4] Wang H, Lambourg E, Guthrie B, Morales DR, Donnan PT, Bell S (2022). Patient outcomes following AKI and AKD: a population-based cohort study. BMC Med.

[5] Huang R, Fu P, Ma L (2023). Kidney fibrosis: from mechanisms to therapeutic medicines. Signal Transduct Target Ther.

[6] Zhu Z, Hu J, Chen Z, Feng J, Yang X, Liang W (2022). Transition of acute kidney injury to chronic kidney disease: role of metabolic reprogramming. Metabolism.

[7] Su L, Zhang J, Gomez H, Kellum JA, Peng Z (2023). Mitochondria ROS and mitophagy in acute kidney injury. Autophagy.

[8] Livingston MJ, Wang J, Zhou J, Wu G, Ganley IG, Hill JA (2019). Clearance of damaged mitochondria via mitophagy is important to the protective effect of ischemic preconditioning in kidneys. Autophagy.

[9] Tang C, Cai J, Yin XM, Weinberg JM, Venkatachalam MA, Dong Z (2021). Mitochondrial quality control in kidney injury and repair. Nat Rev Nephrol.

[10] Ruiz-Ortega M, Rayego-Mateos S, Lamas S, Ortiz A, Rodrigues-Diez RR (2020). Targeting the progression of chronic kidney disease. Nat Rev Nephrol.

[11] Zhu J, Zhang G, Song Z, Xiang X, Shu S, Liu Z (2020). Protein kinase C-delta mediates kidney tubular injury in cold storage-associated kidney transplantation. J Am Soc Nephrol.

[12] Shi L, Song Z, Li Y, Huang J, Zhao F, Luo Y (2023). MiR-20a-5p alleviates kidney ischemia/reperfusion injury by targeting ACSL4-dependent ferroptosis. Am J Transplant.

[13] Zhu J, Xiang X, Hu X, Li C, Song Z, Dong Z (2023). miR-147 represses NDUFA4, inducing mitochondrial dysfunction and tubular damage in cold storage kidney transplantation. J Am Soc Nephrol.

[14] Zhao F, Zhu J, Zhang M, Luo Y, Li Y, Shi L (2023). OGG1 aggravates renal ischemia-reperfusion injury by repressing PINK1-mediated mitophagy. Cell Prolif.

[15] Motwani M, Pesiridis S, Fitzgerald KA (2019). DNA sensing by the cGAS-STING pathway in health and disease. Nat Rev Genet.

[16] Ablasser A, Goldeck M, Cavlar T, Deimling T, Witte G, Röhl I (2013). cGAS produces a 2′-5′-linked cyclic dinucleotide second messenger that activates STING. Nature.

[17] Mitrofanova A, Fontanella A, Tolerico M, Mallela S, Molina David J, Zuo Y (2022). Activation of stimulator of IFN genes (STING) causes proteinuria and contributes to glomerular diseases. J Am Soc Nephrol.

[18] Maekawa H, Inoue T, Ouchi H, Jao TM, Inoue R, Nishi H (2019). Mitochondrial damage causes inflammation via cGAS-STING signaling in acute kidney injury. Cell Rep.

[19] Chung KW, Dhillon P, Huang S, Sheng X, Shrestha R, Qiu C (2019). Mitochondrial damage and activation of the STING pathway lead to renal inflammation and fibrosis. Cell Metab.

[20] Zang N, Cui C, Guo X, Song J, Hu H, Yang M (2022). cGAS-STING activation contributes to podocyte injury in diabetic kidney disease. iScience.

[21] He F, Wu Z, Wang Y, Yin L, Lu S, Dai L (2022). Downregulation of tripartite motif protein 11 attenuates cardiomyocyte apoptosis after ischemia/reperfusion injury via DUSP1-JNK1/2. Cell Biol Int.

[22] Chen Z, Chen Q, Cheng Z, Gu J, Feng W, Lei T (2020). Long non-coding RNA CASC9 promotes gefitinib resistance in NSCLC by epigenetic repression of DUSP1. Cell Death Dis.

[23] Liu Z, Wang J, Dai F, Zhang D, Li W (2023). DUSP1 mediates BCG induced apoptosis and inflammatory response in THP-1 cells via MAPKs/NF-kappaB signaling pathway. Sci Rep.

[24] Blumer S, Fang L, Chen WC, Khan P, Hostettler K, Tamm M (2021). IPF-fibroblast Erk1/2 activity is independent from microRNA cluster 17-92 but can be inhibited by treprostinil through DUSP1. Cells.

[25] Tan Y, Zhang Y, He J, Wu F, Wu D, Shi N (2022). Dual specificity phosphatase 1 attenuates inflammation-induced cardiomyopathy by improving mitophagy and mitochondrial metabolism. Mol Metab.

[26] Ge Y, Wang J, Wu D, Zhou Y, Qiu S, Chen J (2019). lncRNA NR_038323 suppresses renal fibrosis in diabetic nephropathy by targeting the miR-324-3p/DUSP1 axis. Mol Ther Nucleic Acids.

[27] Damman J, Bloks VW, Daha MR, van der Most PJ, Sanjabi B, van der Vlies P (2015). Hypoxia and complement-and-coagulation pathways in the deceased organ donor as the major target for intervention to improve renal allograft outcome. Transplantation.

[28] Wang Y, Wang HM, Zhou Y, Hu LH, Wan JM, Yang JH (2023). Dusp1 regulates thermal tolerance limits in zebrafish by maintaining mitochondrial integrity. Zool Res.

[29] McArthur K, Whitehead LW, Heddleston JM, Li L, Padman BS, Oorschot V (2018). BAK/BAX macropores facilitate mitochondrial herniation and mtDNA efflux during apoptosis. Science.

[30] Shen J, Zhang Y, Yu H, Shen B, Liang Y, Jin R (2016). Role of DUSP1/MKP1 in tumorigenesis, tumor progression and therapy. Cancer Med.

[31] Lu C, Wu B, Liao Z, Xue M, Zou Z, Feng J (2021). DUSP1 overexpression attenuates renal tubular mitochondrial dysfunction by restoring Parkin-mediated mitophagy in diabetic nephropathy. Biochem Biophys Res Commun.

[32] Sheng J, Li H, Dai Q, Lu C, Xu M, Zhang J (2019). DUSP1 recuses diabetic nephropathy via repressing JNK-Mff-mitochondrial fission pathways. J Cell Physiol.

[33] Park S, Lee H, Lee J, Lee S, Cho S, Huh H (2022). RNA-seq profiling of tubulointerstitial tissue reveals a potential therapeutic role of dual anti-phosphatase 1 in glomerulonephritis. J Cell Mol Med.

[34] Lechuga-Vieco AV, Latorre-Pellicer A, Calvo E, Torroja C, Pellico J, Acín-Pérez R (2022). Heteroplasmy of wild-type mitochondrial DNA variants in mice causes metabolic heart disease with pulmonary hypertension and frailty. Circulation.

[35] Zhong W, Rao Z, Xu J, Sun Y, Hu H, Wang P (2022). Defective mitophagy in aged macrophages promotes mitochondrial DNA cytosolic leakage to activate STING signaling during liver sterile inflammation. Aging Cell.

[36] Li J, Sun X, Yang N, Ni J, Xie H, Guo H (2023). Phosphoglycerate mutase 5 initiates inflammation in acute kidney injury by triggering mitochondrial DNA release by dephosphorylating the pro-apoptotic protein Bax. Kidney Int.

[37] Zhang Q, Raoof M, Chen Y, Sumi Y, Sursal T, Junger W (2010). Circulating mitochondrial DAMPs cause inflammatory responses to injury. Nature.

[38] Bi X, Du C, Wang X, Wang XY, Han W, Wang Y (2021). Mitochondrial damage-induced innate immune activation in vascular smooth muscle cells promotes chronic kidney disease-associated plaque vulnerability. Adv Sci (Weinh).

[39] Wancket LM, Frazier WJ, Liu Y (2012). Mitogen-activated protein kinase phosphatase (MKP)-1 in immunology, physiology, and disease. Life Sci.

[40] Ramkissoon A, Chaney KE, Milewski D, Williams KB, Williams RL, Choi K (2019). Targeted inhibition of the dual specificity phosphatases DUSP1 and DUSP6 suppress MPNST growth via JNK. Clin Cancer Res.

[41] Guo F, Zhang C, Wang F, Zhang W, Shi X, Zhu Y (2020). Deubiquitinating enzyme USP33 restrains docetaxel-induced apoptosis via stabilising the phosphatase DUSP1 in prostate cancer. Cell Death Differ.

[42] Papadakis ES, Finegan KG, Wang X, Robinson AC, Guo C, Kayahara M (2006). The regulation of Bax by c-Jun N-terminal protein kinase (JNK) is a prerequisite to the mitochondrial-induced apoptotic pathway. FEBS Lett.

[43] Lei K, Davis RJ (2003). JNK phosphorylation of Bim-related members of the Bcl2 family induces Bax-dependent apoptosis. Proc Natl Acad Sci USA.

[44] Tsuruta F, Sunayama J, Mori Y, Hattori S, Shimizu S, Tsujimoto Y (2004). JNK promotes Bax translocation to mitochondria through phosphorylation of 14-3-3 proteins. EMBO J.

[45] White MJ, McArthur K, Metcalf D, Lane RM, Cambier JC, Herold MJ (2014). Apoptotic caspases suppress mtDNA-induced STING-mediated type I IFN production. Cell.

[46] Jin Q, Li R, Hu N, Xin T, Zhu P, Hu S (2018). DUSP1 alleviates cardiac ischemia/reperfusion injury by suppressing the Mff-required mitochondrial fission and Bnip3-related mitophagy via the JNK pathways. Redox Biol.

