# Supplementary material for: DUSP1 protects against ischemic acute kidney injury through stabilizing mtDNA via interaction with JNK
Source: Cell Death Dis. 2023 Nov 7;14(11):724. doi: 10.1038/s41419-023-06247-4 (PMC10630453; doi:10.1038/s41419-023-06247-4)
Supplement: Supplementary file 1 — Supplementary materials [file 41419_2023_6247_MOESM1_ESM.doc]

**Supplementary materials**

**Supplementary Figure 1**

|  |
| --- |
| Supplementary Figure 1. Representative microscopy images of JC-1 staining; red fluorescence represents the mitochondrial aggregate JC-1 and green fluorescence indicates the monomer JC-1. Quantification of the mitochondrial membrane potential. *p<0.05 versus respective control group . #, p < 0.05 versus pc-DNA3.1-H_2_O_2_ group. (n = 5). |

**Supplementary Figure 2**

|  |
| --- |
| Supplementary Figure 2. Analysis and visualization of two-dimensional angles of action forces between Dusp1 protein and JNK protein.  The 259 th TYR amino acid residue and the 263rd ARG amino acid residue of the JNK protein can bind to the 214th GLU amino acid residue of the dusp1 protein through two hydrogen bonds with lengths of 3.12Å and 1.05Å, respectively; the 156th ARG amino acid residue of the Dusp1 protein can bind to the 329th GLU amino acid residue of the JNK protein through two hydrogen bonds with lengths of 2.86Å and 2.64Å, respectively. In the two-dimensional diagram, green dashed lines represent hydrogen bonds and red dashed lines represent hydrophobic interactions. |
