# Supplementary material for: DUSP1 protects against ischemic acute kidney injury through stabilizing mtDNA via interaction with JNK
Source: Cell Death Dis. 2023 Nov 7;14(11):724. doi: 10.1038/s41419-023-06247-4 (PMC10630453; doi:10.1038/s41419-023-06247-4)
Supplement: Supplementary file 4 — Editing Certificate [file 41419_2023_6247_MOESM4_ESM.pdf]

EDITSPRINGS

EDITORIAL CERTIFICATE

This is to certify that the manuscript detailed below was edited by one or more of our highly qualified, native English speakers at EditSprings, to assure compliance with Anglophone academic standards in terms of style, punctuation, grammar, and spelling.

Manuscript title:

**DUSP1 protects against ischemic acute kidney injury through stabilizing mtDNA via interact
ion with JNK**

Authors:

Lang Shi#, Hongchu Zha#, Zhou Pan, Jiayi Wang, Yao Xia, Huiming Li, Hua Huang, Ruchi Yue, Zhixia Song, Jiefu Zhu*

Date Issued:

Aug 03 2023

Certificate Number:

ES-202211301048876512

Editing Certified * EditSprings * EditSprings * EditSprings
Edit Springs

This certificate can be verified on https://www.editsprings.com/QueryCertificate.html . EditSprings hereby certifies that neither content nor the author's intentions were altered in any way during the editing process. Documents in receipt of this certification should be ready for publication as far as style and language are concerned, provided that the author(s) accepted our suggestions and changes (which remains their right and responsibility).

EditSprings offers a wide range of editing, translation, for researchers and publishers across the world. Our highly skilled editors are all established academics based in Anglophone Higher Education institutions across the world (U.K., U.S.A., Canada, Australia, and elsewhere), are experts in their respective fields, and are qualified to edit research papers authored by non-Anglophone scholars.
