# Supplementary material for: DUSP1 protects against ischemic acute kidney injury through stabilizing mtDNA via interaction with JNK
Source: Cell Death Dis. 2023 Nov 7;14(11):724. doi: 10.1038/s41419-023-06247-4 (PMC10630453; doi:10.1038/s41419-023-06247-4)
Supplement: Supplementary file 2 — Authorship Change Approval [file 41419_2023_6247_MOESM2_ESM.pdf]

Dear editor,

During the revision phase, we decided to include an additional author, "Jiayi Wang," to our manuscript. We believe that their contributions to the research are valuable and deserve appropriate acknowledgment.

Below are the details of the article:

Title: DUSP1 protects against ischemic acute kidney injury through stabilizing mtDNA via interaction with JNK

Authors: [Lang Shi[#], Hongchu Zha[#], Zhou Pan, Jiayi Wang, Yao Xia, Huimin Li, Hua Huang, Ruchi Yue, Zhixia Song, Jiefu Zhu* (please include Jiayi Wang)]

yijia wang， Department of Anesthesiology, The Second Xiangya Hospital, Central South University, Changsha, China ，Email:168202068@csu.edu.cn

*Corresponding Author: Jiefu Zhu

[#] Lang Shi and Hongchu Zha contributed equally.

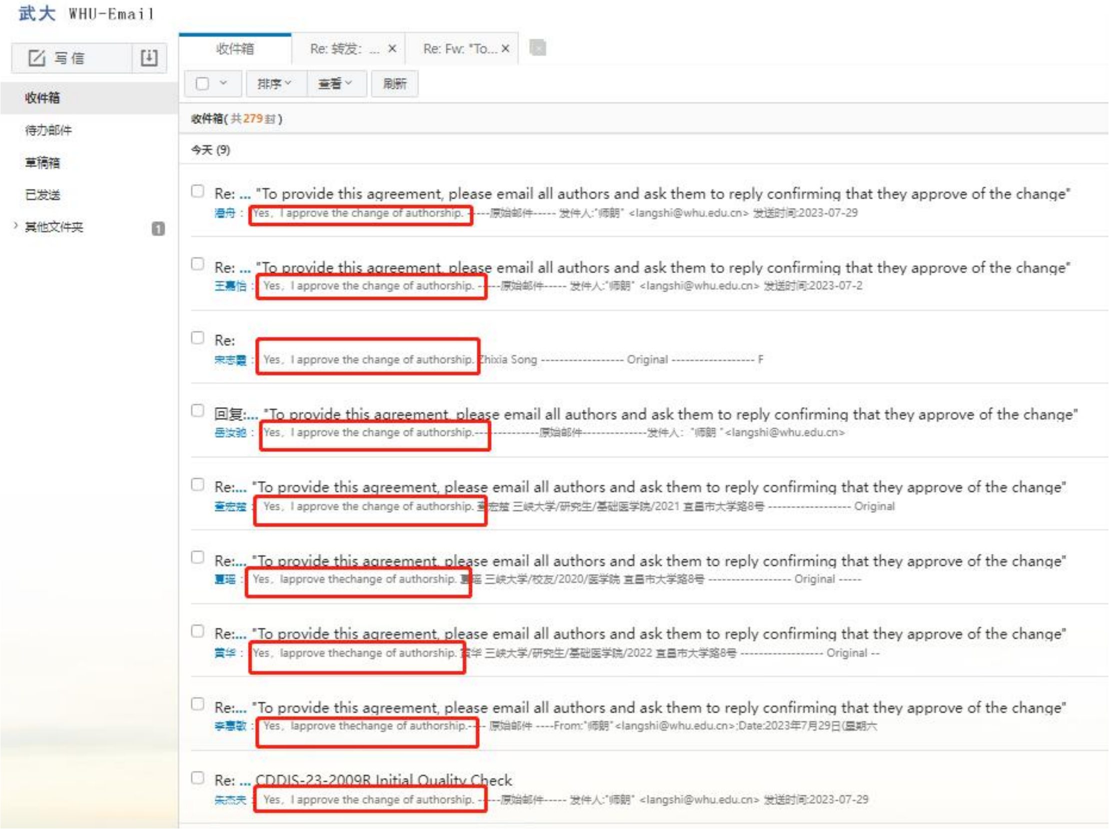

武大 WHU-Email

写信

收件箱
待办邮件
草稿箱
已发送
其他文件夹

欢迎页 | Re: Fw: "To...

回复 | 回复全部 | 转发 | 移动到 | 标记为 | 更多 | 删除

Re: Fw: "To provide this agreement, please email all authors and ask them to reply confirming that they approve of the c

发件人： "潘舟" <zhou.pan1156@whu.edu.cn>
收件人： "师朗" <langshi@whu.edu.cn>
抄 送： "yijia wang" <168202068@csu.edu.cn> "zhahongchu" <zhahongchu@ctgu.edu.cn> "yao xia" <xiayao@ctgu.edu.cn> "lihuimin" <lihuimin@ctgu.edu.cn> "hua h

Yes, I approve the change of authorship.

------原始邮件------
发件人:"师朗" <langshi@whu.edu.cn>
发送时间:2023-07-29 16:12:58 (星期六)
收件人: "yijia wang" <168202068@csu.edu.cn>, zhahongchu <zhahongchu@ctgu.edu.cn>, "zhou pan" <zhou.pan1156@whu.edu.cn>, "yao xia" <xiayao@ctgu.edu.cn>,
抄送:
主题: Fw: "To provide this agreement, please email all authors and ask them to reply confirming that they approve of the change"

------原始邮件------
发件人:langshi@whu.edu.cn
发送时间:2023-07-29 16:02:54 (星期六)
收件人: "yijia wang" <168202068@csu.edu.cn>, zhahongchu <zhahongchu@ctgu.edu.cn>, "zhou pan" <zhou.pan1156@whu.edu.cn>, "yao xia" <xiayao@ctgu.edu.cn>,
抄送:
主题:

—— 转发的邮件 ——

武大 WHU-Email

写信

收件箱
待办邮件
草稿箱
已发送
其他文件夹

收件箱 | Re: 转发: ... | Re: Fw: "To... | Re:

回复 | 回复全部 | 转发 | 移动到 | 标记为 | 更多 | 删除

Re:

发件人： "宋志霞" <songzhixia@ctgu.edu.cn>
收件人： "师朗" <langshi@whu.edu.cn>

Yes, I approve the change of authorship.

Zhixia Song

------------------ Original ------------------
From: "师朗"<langshi@whu.edu.cn>;
Date: Sat, Jul 29, 2023 04:02 PM
To: "yijia wang"<168202068@csu.edu.cn>; "zhahongchu"<zhahongchu@ctgu.edu.cn>; "zhou pan"<zhou.pan1156@whu.edu.cn>; "yao xia"<xiayao@
<songzhixia@ctgu.edu.cn>;
Subject:

—— 转发的邮件 ——

武大 WHU-Email

写信

收件箱
待办邮件
草稿箱
已发送
其他文件夹

收件箱 | Re: 转发: ... | Re: Fw: "To...

回复 | 回复全部 | 转发 | 移动到 | 标记为 | 更多 | 删除

Re: Fw: "To provide this agreement, please email all authors and ask them to reply confirming that they approve

发件人： "王嘉怡" <168202068@csu.edu.cn>
收件人： "师朗" <langshi@whu.edu.cn>

Yes, I approve the change of authorship.

——原始邮件——
发件人:"师朗" <langshi@whu.edu.cn>
发送时间:2023-07-29 16:12:58 (星期六)
收件人: "yijia wang" <168202068@csu.edu.cn>, zhahongchu <zhahongchu@ctgu.edu.cn>, "zhou pan" <zhou.pan1156@whu.edu.cn>, "yao xia" <xiayao@ctgu
chi" <yueruchi@ctgu.edu.cn>, "zhixia song" <songzhixia@ctgu.edu.cn>
抄送:
主题: Fw: "To provide this agreement, please email all authors and ask them to reply confirming that they approve of the change"

武大 WHU-Email

写信

收件箱 | Re: 转发: … × | Re: Fw: "To… × | Re: × | 回复:Fw: "T… ×

回复 回复全部 转发 移动到 标记为 更多 删除

收件箱
待办邮件
草稿箱
已发送
其他文件夹

回复:Fw: "To provide this agreement, please email all authors and ask them to reply confirming that they approve

发件人： "岳汝驰" <yueruchi@ctgu.edu.cn>

收件人： "师朗" <langshi@whu.edu.cn> "yijia wang" <168202068@csu.edu.cn> "zhahongchu" <zhahongchu@ctgu.edu.cn> "zhou pan" <zhou.pan1156@whu.edu.

Yes, I approve the change of authorship.

——————原始邮件——————
发件人: "师朗 "<langshi@whu.edu.cn>;
发送时间: 2023年7月29日(星期六) 下午4:13
收件人: "yijia wang" <168202068@csu.edu.cn>;"zhahongchu" <zhahongchu@ctgu.edu.cn>;"zhou pan" <zhou.pan1156@whu.edu.cn>;"yao xia" <xiayao@ctgu.edu
<yueruchi@ctgu.edu.cn>;"zhixia song" <songzhixia@ctgu.edu.cn>;
主题: Fw: "To provide this agreement, please email all authors and ask them to reply confirming that they approve of the change"

原始邮件

武大 WHU-Email

写信

收件箱 | Re: 转发: … × | Re: Fw: "To… × | Re: × | 回复:Fw: "T… × | Re:Fw: "To … ×

回复 回复全部 转发 移动到 标记为 更多 删除

收件箱
待办邮件
草稿箱
已发送
其他文件夹

Re:Fw: "To provide this agreement, please email all authors and ask them to reply confirming that they approv

发件人： "查宏楚" <zhahongchu@ctgu.edu.cn>

收件人： "师朗" <langshi@whu.edu.cn>

Yes, I approve the change of authorship.

Mail 三峡飞鸿

查宏楚
三峡大学/研究生/基础医学院/2021
宜昌市大学路8号

武大 WHU-Email

写信

收件箱 | Re: 转发: … × | Re: Fw: "To… × | Re: × | 回复:Fw: "T… × | Re:Fw: "To … × | Re:Fw: "To …

回复 回复全部 转发 移动到 标记为 更多 删除

收件箱
待办邮件
草稿箱
已发送
其他文件夹

Re:Fw: "To provide this agreement, please email all authors and ask them to reply confirming

发件人： "夏瑶" <xiayao@ctgu.edu.cn>

收件人： "师朗" <langshi@whu.edu.cn>

Yes, lapprove thechange of authorship.

Mail 三峡飞鸿

夏瑶
三峡大学/校友/2020/医学院
宜昌市大学路8号

武大 WHU-Email

写信

收件箱 | Re: 转发: ... × | Re: Fw: "To... × | Re: × | 回复:Fw: "T... × | Re:Fw: "To ... × | Re:Fw: "To ...

收件箱
待办邮件
草稿箱
已发送
› 其他文件夹

回复 | 回复全部 | 转发 | 移动到 ˅ | 标记为 ˅ | 更多 ˅ | 删除

Re:Fw: "To provide this agreement, please email all authors and ask them to reply confirming

发件人： "李惠敏" <lihuimin@ctgu.edu.cn>

收件人： "langshi" <langshi@whu.edu.cn> "168202068" <168202068@csu.edu.cn> "zhahongchu" <zhahongchu@ctgu.edu.cn> "zho

Yes, lapprove thechange of authorship.

—— 原始邮件 ——

From:"师朗"<langshi@whu.edu.cn>;

Date:2023年7月29日(星期六) 下午4:12

To:"yijia wang"<168202068@csu.edu.cn>;"zhahongchu"<zhahongchu@ctgu.edu.cn>;"zhou pan"<zhou.pan1156@whu.edu.cn>;"yao x <yueruchi@ctgu.edu.cn>;"zhixia song"<songzhixia@ctgu.edu.cn>;

Subject:Fw: "To provide this agreement, please email all authors and ask them to reply confirming that t

---

武大 WHU-Email

写信

收件箱 | Re: 转发: ... × | Re: Fw: "To... × | Re: × | 回复:Fw: "T... × | Re:Fw: "To ... × | Re:Fw: "To ...

收件箱
待办邮件
草稿箱
已发送
› 其他文件夹

回复 | 回复全部 | 转发 | 移动到 ˅ | 标记为 ˅ | 更多 ˅ | 删除

Re:Fw: "To provide this agreement, please email all authors and ask them to reply confirming

发件人： "黄华" <huang.hua@ctgu.edu.cn>

收件人： "师朗" <langshi@whu.edu.cn>

Yes, lapprove thechange of authorship.

Mail 三峡飞鸿

黄华
三峡大学/研究生/基础医学院/2022
宜昌市大学路8号
